# Whole Genome Profiling of Lung Microbiome in Solid Organ Transplant Recipients Reveals Virus Involved Microecology May Worsen Prognosis

**DOI:** 10.3389/fcimb.2022.863399

**Published:** 2022-03-16

**Authors:** Lingai Pan, Fengsheng Wu, Qingqing Cai, Zhuofei Xu, Huan Hu, Tian Tang, Ruiming Yue, Yifu Hou, Xiaoqin Zhang, Yuan Fang, Xiaobo Huang, Yan Kang

**Affiliations:** ^1^ Department of Critical Care Medicine, West China Hospital, West China Clinical Medical School, Sichuan University, Chengdu, China; ^2^ Department of Critical Care Medicine, Sichuan Provincial People’s Hospital, University of Electronic Science and Technology of China, Chengdu, China; ^3^ Genoxor Medical Science and Technology Inc., Zhejiang, China; ^4^ Department of Organ Transplant Center, Sichuan Provincial People’s Hospital, University of Electronic Science and Technology of China, Chengdu, China

**Keywords:** solid organ transplant, virus, mortality, lung microbiome, cytomegalovirus

## Abstract

Solid organ transplantation (SOT) is the final therapeutic option for recipients with end-stage organ failure, and its long-term success is limited by infections and chronic allograft dysfunction. Viral infection in SOT recipients is considered an important factor affecting prognosis. In this study, we retrospectively analyzed 43 cases of respiratory infections in SOT recipients using metagenomic next-generation sequencing (mNGS) for bronchoalveolar lavage fluid (BALF). At least one virus was detected in 26 (60.5%) recipients, while 17 (39.5%) were virus-negative. Among virus-positive recipients, cytomegalovirus (CMV) was detected in 14 (32.6%), Torque teno virus (TTV) was detected in 9 (20.9%), and other viruses were detected in 6 (14.0%). Prognostic analysis showed that the mortality of the virus-positive group was higher than that of the virus-negative group regardless whether it is the main cause of infection. Analysis of different types of viruses showed that the mortality of the CMV-positive group was significantly higher than that of the CMV-negative group, but no significant difference was observed in other type of virus groups. The diversity analysis of the lung microbiome showed that there was a significant difference between the virus-positive group and the negative group, in particular, the significant differences in microorganisms such as *Pneumocystis jirovecii* (PJP) and *Moraxella osloensis*were detected. Moreover, in the presence of CMV, *Pneumocystis jirovecii*, *Veillonella parvula*, and other species showed dramatic changes in the lung of SOT patients, implying that high degree of co-infection between CMV and *Pneumocystis jirovecii may occur*. Taken together, our study shows that the presence of virus is associated with worse prognosis and dramatically altered lung microbiota in SOT recipients.

## Introduction

Solid organ transplantation (SOT) is the final therapeutic option for recipients with end-stage organ failure, and its long-term success is limited by infections and chronic allograft dysfunction (Timsit et al., 2019). For lung transplantation, infections and allograft dysfunction problems account for 25% - 30% of mortality during the first year post-transplant (Trulock et al., 2007). Because of their immunocompromised state, SOT recipients are at high risk for viral infections, which are a major complication post-transplant and continue to be a potential contributor to graft failure or cause of severe mortality (Fishman, 2007). Recent research found the association of respiratory viruses such as respiratory syncytial virus (RSV), parainfluenza virus, and influenza viruses with increased morbidity following transplant (Bailey et al., 2019).

Cytomegalovirus (CMV), the most common viral pathogen, historically has been associated with worse mortality in SOT recipients (Razonable, 2010). Recent antiviral drugs have improved treatment and prophylaxis of CMV infection. However, other viruses have been recently recognized as having a potential role in affecting the outcome in the SOT recipients (Zanella et al., 2020). The use of immunosuppressive therapies may change the viral spectrum, and the immunopathological mechanisms of viral infection in SOT recipients remain incompletely understood. Sensitive and comprehensive methods to detect the viral pathogen are essential for diagnosis because of the varying immunocompromised status of the hosts, and sometimes the clinical syndromes are nonspecific. Metagenomic next-generation sequence (mNGS) with rapid turnaround times greatly improved the ability to detect the viral infections in a timely fashion.

In this study, we aimed to comprehensively examine the real-world clinical impact of virus on the outcome in SOT recipients. Bronchoalveolar lavage fluid (BALF) samples were collected from SOT recipients with lung infections for mNGS. According to the results of mNGS, we analyzed the prognostic difference between the virus-positive group and the negative group and studied the contribution of different viruses to the prognosis. In addition, we analyzed the impact of virus on the microbiome of the lungs. Our results indicate that the virus is associated with a worse prognosis, whether or not it is the main cause of infection. Our results may have important implications for the clinical management and follow-up research in SOT recipients.

## Materials and Methods

### Recipient Enrollment and Specimen Collection

The solid organ transplant recipients included in this study were all from Sichuan Provincial People’s Hospital. Our study passed the review of the Medical Ethics Committee of Sichuan Provincial People’s Hospital, and each enrolled recipient or their family signed a written informed consent. From November 2018 to September 2020, recipients with suspected infection were enrolled in this study following kidney, lung, or liver transplantation, and the recipients’ alveolar lavage fluid was collected from suspected lung infection recipients for mNGS analysis. The diagnosis of lung infection is based on the following: (1) There are related clinical symptoms, such as cough and sputum, difficulty breathing, fever, etc.; (2) There are related manifestations in imaging, such as pulmonary exudation; (3) Two or more SOT experts highly suspect lung infection with other content. Data collected on the enrolled recipients included body mass index (BMI), ICU length of stay, sequential organ failure assessment (SOFA) and Acute Physiology and Chronic Health Evaluation (APACHE II) scores, chronic disease history, and findings on other routine examinations.

### Sample Processing and mNGS Detection

Samples were taken from recipients in two situations: BALF was collected when lung infection occurred during hospitalization, and β-lactam or carbapenem antibiotics were used for prophylactic treatment; when lung infection occurred after discharge, BALF was collected before anti-infective treatment. Samples of 1.5-3 mL BALF were collected according to standard procedures. A 1.5- mL microcentrifuge tube with 0.6-mL sample, enzyme, and 1 g of 0.5 mm glass beads was attached to a horizontal platform on a vortex mixer and agitated vigorously at 2800-3200 rpm for 30 min. Then the 0.3-mL sample was separated into new 1.5-mL microcentrifuge tubes and DNA was extracted using the TIANamp Micro DNA Kit (DP316, Tiangen Biotech) according to the manufacturer’s instructions. Then, DNA libraries were constructed through DNA fragmentation, end repair, adapter ligation, and PCR amplification. Agilent 2100 was used for quality control of the DNA libraries. Quality-qualified libraries were sequenced on the BGISEQ-50/MGISEQ-2000 platform. High-quality sequencing data were generated by removing low-quality reads, followed by computational subtraction of human-host sequences mapped to the human reference genome (hg19) using Burrows-Wheeler Alignment. After removal of low-complexity reads, the remaining data were classified by simultaneous alignment with four microbial genome databases, consisting of bacteria, fungi, viruses, and parasites.

### Data Analysis

#### Survival Proportions

The survival of recipients within 90 days after transplantation was recorded, and the survival curve was made using GraphPad Prism 9. Comparison of survival curves was performed by the log-rank (Mantel-Cox) test.

#### Alpha Diversity

The analysis of alpha diversity was used to estimate complexity of taxonomic diversity for individual samples based on the Shannon index. The data matrix consisting of relative abundance for individual taxa across the samples was used for the Shannon index boxplot using R packages ggplot2 v3.3.2 and ggpubr v0.4.0. The visualization was implemented in the R environment v4.03.

#### Beta Diversity (NMDS)

To assess the compositional similarity among the studied samples from different microbial communities, the Bray-Curtis measure of beta diversity was employed to compare all pairwise taxonomic relative abundances using an R function vegdist in the package vegan v2.5-7. Based on the resulting Bray-Curtis similarity distance matrix, non-metric multidimensional scaling (NMDS) was adopted to display the dispersion of community structure using an R function metaMDS in the package vegan v2.5-7. The scatterplot was implemented by using ggplot2 v3.3.2 in the R environment v4.03.

#### Beta Diversity (PCoA)

To assess the compositional similarity among the studied samples from different microbial communities, the Bray-Curtis measure of beta diversity was employed to compare all pairwise taxonomic relative abundances using an R function vegdist in the package vegan v2.5-7. Based on the resulting Bray-Curtis similarity distance matrix, Principal Coordinate Analysis (PCoA) was adopted to display the dispersion of community structure using an R function cmdscale in the package vegan v2.5-7. The scatterplot was implemented by using ggplot2 v3.3.2 in the R environment v4.03.

#### Differential Analysis Between Groups of Microbial Communities

The statistical difference for the taxonomic profiles between study groups was evaluated using the function ANOSIM from the R package vegan v2.5-7. The similarity boxplot between groups was implemented using R package ggplot2 v3.3.2.

#### Heatmap of Microbial Community Structure

Based on the taxonomic relative abundance profile, the heatmap for visualizing relative abundances of the top 20 taxa was created using pheatmap v1.0.12 in the R environment v4.03.

#### Relative Abundance Boxplot of the Top 20 Taxa

Based on the taxonomic relative abundance profile, the boxplot of the top 20 taxa is created using ggplot2 v3.3.2 in the R environment v4.03.

## Results

### Enrollment Information of Solid Organ Transplant Recipients

**Figure 1:**
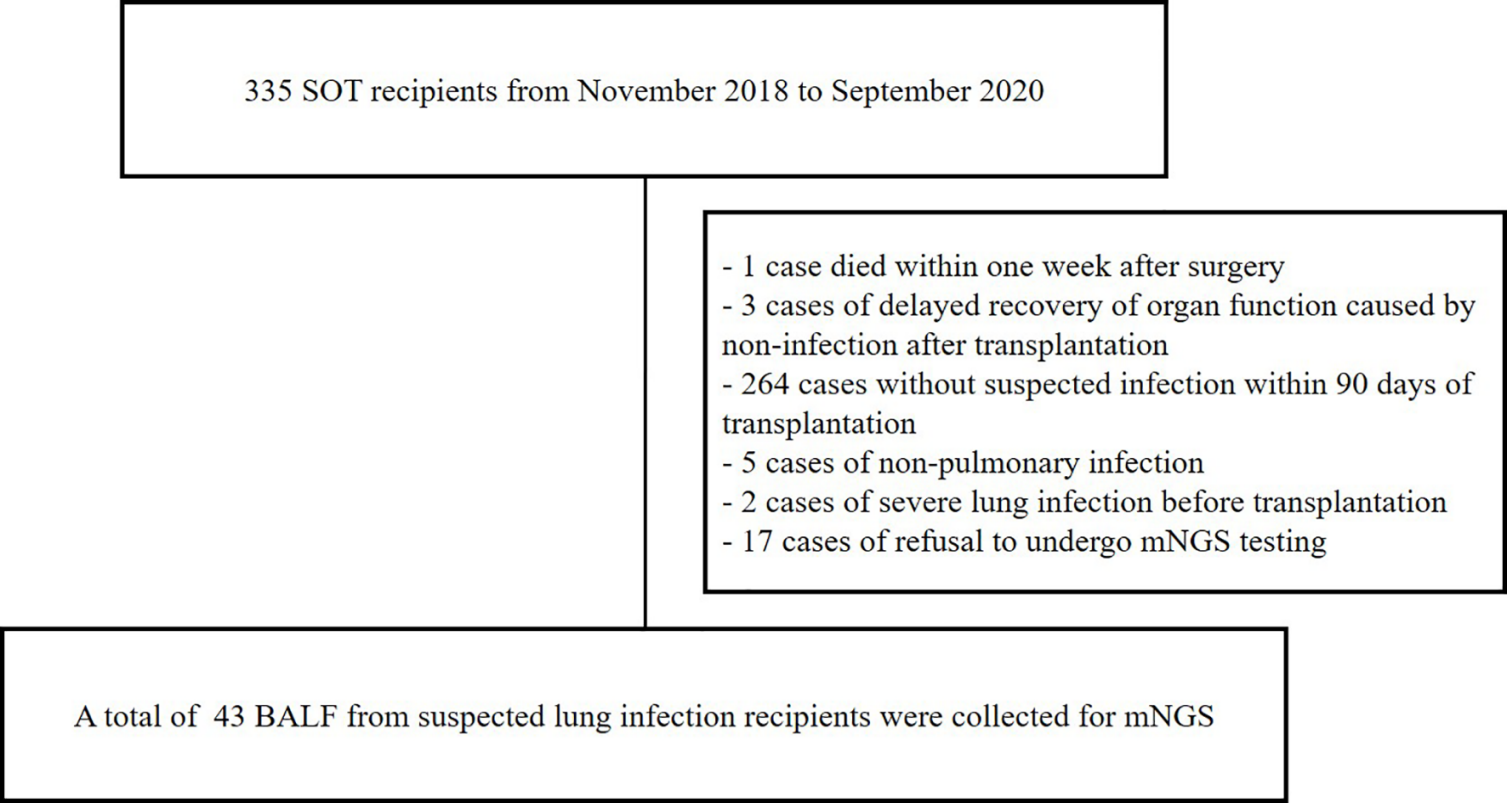
Study design in solid organ transplantation.

A total of 43 SOT recipients were enrolled in this study (**Figure 1**) , including 26 with kidney transplants, 13 with lung transplants, and 4 with liver transplants. The average age of all recipients was 52 years old, 31 were males, and the average BMI was 20.8 kg/m^2^. Of which, 32 recipients have been admitted to the ICU, average ICU stay was 8.8 days. There were 32 recipients evaluated by SOFA and APACHE II with average scores of 6.7 and 10.9, respectively. The most frequent chronic disease among the recipients was hypertension (17 cases), followed by hepatitis B viral infection (8 cases), diabetes, chronic obstructive pulmonary disease, and coronary heart disease (**Table 1**). The information of all recipients is listed in **Supplementary Table 1**.

**Table 1:** Demographic and Clinical Characteristics of the 43 Recipients with solid organ transplantation.

Characteristic	Value
Mean age, y	52
Male	31
BMI, kg/m^2^	20.8
**Severity of illness**	
Mean SOFA	6.7
Mean APACHE II	10.9
**Previous history of chronic disease-no. (%)**	
HBV	4 (9.3)
HBV and HTN	3 (7.0)
HBV, HTN and DM	1 (2.3)
HTN	12 (27.9)
HTN and COPD	1 (2.3)
TB	1 (2.3)
TB and CHD	1 (2.3)
Chronic bronchitis	1 (2.3)
DM	1 (2.3)
Uremia	1 (2.3)
Non	17 (39.5)
**Type of solid organ transplantation**	
Kidney*	26
Lung	13
Liver^	4
**ICU outcomes**	
Mean ICU length of stay, days	8.8

BMI, Body Mass Index; SOFA, Sequential Organ Failure Score; APACHE II, Acute Physiology and Chronic Health Score II; HBV, Hepatitis B Virus; HTN, Hypertension; DM, Diabetes mellitus; COPD, Chronic obstructive pulmonary disease; CHD, Coronary heart disease.

*1 recipient underwent a second renal transplant. ^1 recipient underwent a second liver transplant. BALF of the two recipients were collected after the first transplant with lung infection.

### Virus Detected in Respiratory Sample of Transplant Recipients Is Associated With Increased Mortality

**Figure 2:**
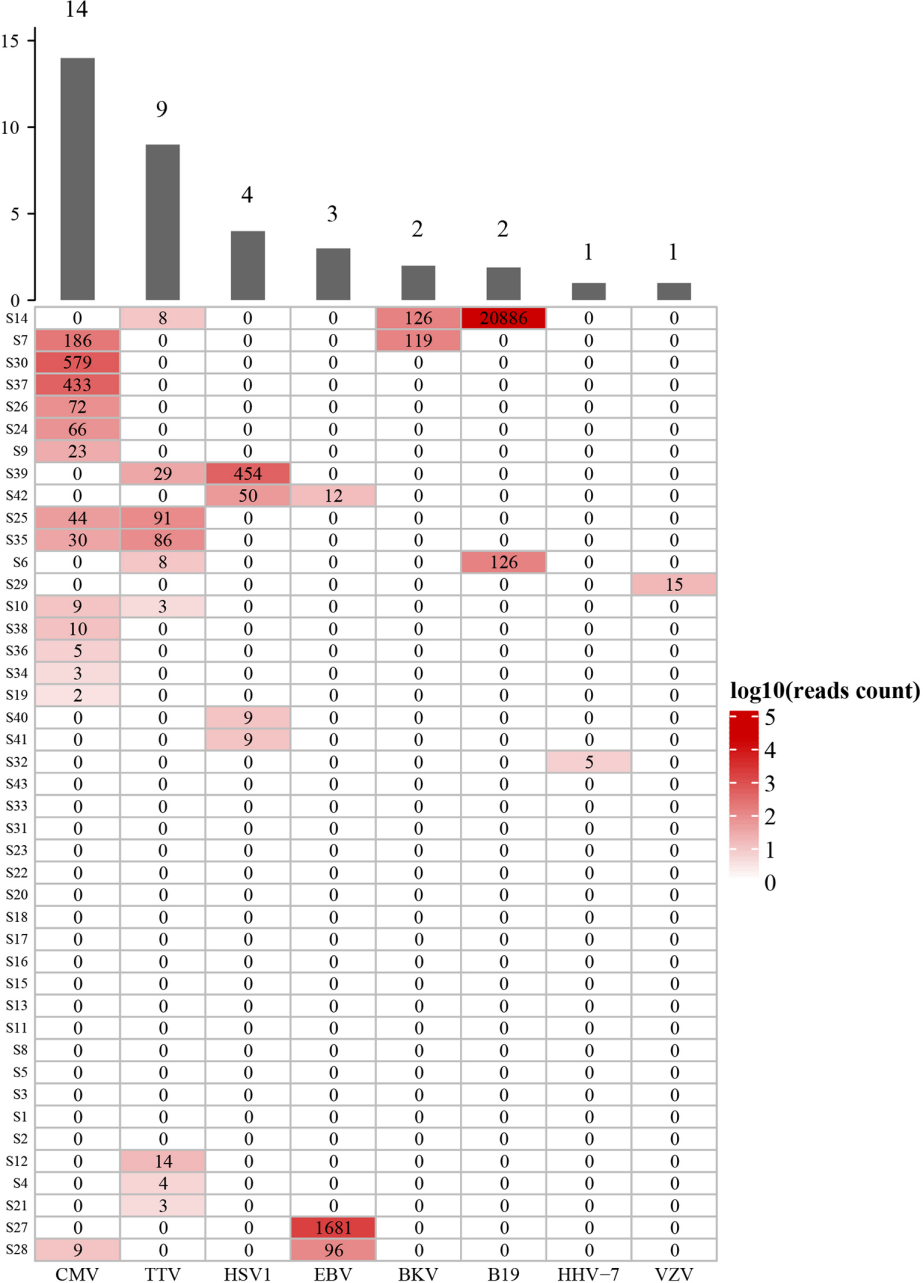
Diversity and reads counts of viruses identified in this study. CMV, Human betaherpesvirus 5; TTV, Torque teno virus; HSV1, Human alphaherpesvirus 1; EBV, Human gammaherpesvirus 4; BKV, BK virus; B19, Human erythroparvovirus 19; HHV-7, Human betaherpesvirus 7; VZV, Varicella Zoster Virus. The numbers on the histogram represent the sample sizes of the corresponding virus. The numbers in the table represent the reads of mNGS results. S14 and S7 etc, sample number. The samples are sorted according to hierarchical clustering with Euclidean distance.

The mNGS in bronchoalveolar lavage fluid (BALF) samples was performed in all SOT recipients to study virus and other microbial communities. At least one virus was detected in 26 (60.5%) recipients, while 17 (39.5%) were virus-negative. Among virus-positive recipients, cytomegalovirus (CMV) was detected in 14 (32.6%) recipients, and TTV was detected in 9 (20.9%) recipients, and other viruses were detected in 6 (14.0%) recipients, as shown in **Figure 2**. Moreover, we analyzed the viral profiles of the three transplant types individually. Interestingly, the results showed that CMV was more frequently detected in kidney transplantation, and the both two BKV-positive cases were detected in kidney transplantation, as shown in **Supplementary Table 3**.

**Figure 3:**
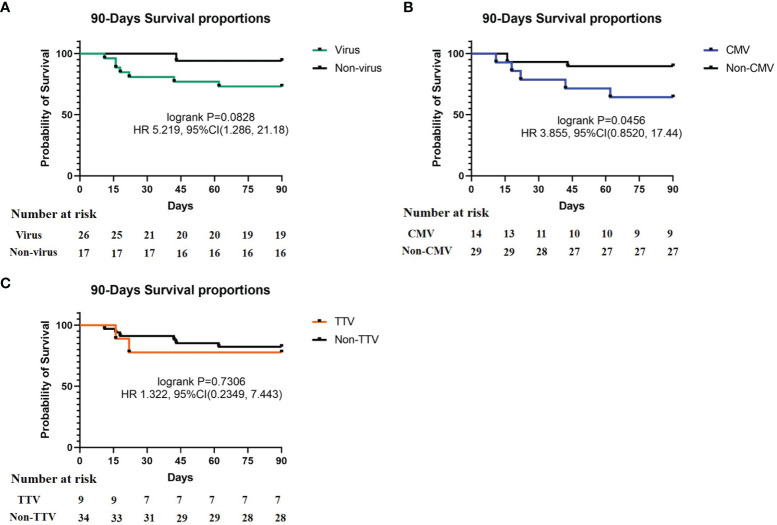
90-Days Survival proportions of different virus. **(A)** Survival proportions of virus and non-virus, p = 0.0828; **(B)** survival proportions of CMV and non-CMV, p = 0.0456; **(C)** survival proportions of TTV and non-TTV, p = 0.7306. Recipient number is displayed in the Number at risk.

To observe the impact of virus on prognosis in these recipients, we evaluated the severity of disease (SOFA and APACHE II scores) and mortality (whether the recipient survived 90 days after transplantation). The results showed that there was no significant difference in SOFA and APACHE II scores between the virus-positive group and the virus-negative group. In this study, a total of 8 cases died within 90 days after transplantation, all of which were related to infection. However, in some cases, the virus may not be considered as the main cause of infection (**Supplementary Table 1**). The 90-days mortality of the virus-positive group was higher than that of the virus-negative group (26.9% vs. 5.9%). In order to explore the impact and contribution of different viruses to the increasing mortality, we separately analyzed the mortality of the CMV-positive group, TTV- positive group, and other virus-positive groups along with mortality in the CMV- and TTV-negative groups). The results showed that the prognosis in the CMV-positive group was significantly worse than that in the CMV-negative group (mortality 35.7% vs. 10.3%, respectively). The mortality of the TTV-positive group (22.2% vs. 17.6%) and the other virus group (18.2% vs. 18.8%) was similar to that of the corresponding negative group (**Table 2**). The survival curves of the virus-positive group and the virus-negative group were significantly separated, but the difference between the two groups was not significant (p = 0.0828). In single-virus groups, the curves of CMV-positive and CMV-negative recipients were significantly separated, and the difference was statistically significant (p = 0.0456). There was no significant difference between TTV-positive and TTV-negative groups (**Figure 3**). At the same time, we analyzed the impact of virus on the mortality of different types of SOT recipients. The data showed that the results of kidney and lung transplantation were similar, and the mortality of liver transplantation was not statistically significant due to the small number of samples (**Supplementary Table 2**). Our results indicated that virus detected in respiratory sample was associated with increased mortality of SOT recipients, and CMV may be the main cause.

**Table 2:** Demographic and Clinical Characteristics of study cohort.

Severity of illness	Virus	Non-virus	P-value
Mean SOFA	6.8	6.5	0.77
Mean APACHE II	10.7	11.1	0.53
**The Prognosis of 90 days-no. (%)**			
Good	19 (73.1%)	16 (94.1%)	0.083
Poor	7 (26.9%)	1 (5.9%)	
**Severity of illness**	**CMV**	**Non-CMV**	**P-value**
Mean SOFA	6.6	6.7	0.98
Mean APACHE II	11.6	10.6	0.52
**The Prognosis of 90 days-no. (%)**			
Good	9 (64.3%)	26 (89.7%)	0.046
Poor	5 (35.7%)	3 (10.3%)	

Good=survival; Poor=died.

### Virus Affects the Lung Microbiome of Solid Organ Transplant Recipients

**Figure 4:**
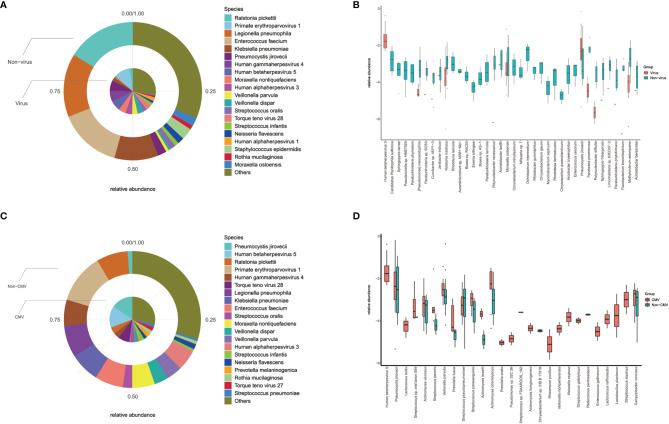
Differences of lung microbiota in groups. **(A)** Top 20 most abundant species and their relative abundance in virus and non-virus recipients; **(B)** there were significant differences in top-20 species (such as *Pneumocystis jirovecii, Moraxella osloensis*, CMV) and other non-top species between virus and non-virus group; **(C)** top 20 most abundant species and their relative abundance in CMV and non-CMV infectious recipients; **(D)** there were significant differences in top-20 species (such as *Pneumocystis jirovecii, Veillonella parvula*) and other non-top species between CMV and non-CMV group.

The mNGS results of all recipients can reflect the status of their lung microbial communities. We analyzed the same amount of random data from all samples to explore whether there are differences in the lung microbiome among these recipients. The diversity analysis of the community structure showed that there was a significant difference between the virus-positive group and the virus-negative group, and analysis showed that the difference between the groups was greater than that within the group, as shown in **Supplementary Figure 1**. A further comparison of species of microorganisms in the virus-positive group and the virus-negative group showed significant differences in the top 20 lung species (such as *Pneumocystis jirovecii*, *Moraxella osloensis*, CMV) and other species beyond the top 20 (**Figure 4**). These results indicate that the virus affects the composition of lung microbial species in solid organ transplant recipients.

Considering that CMV is the main type of virus in our study (14/26) and its impact on mortality, we did a similar analysis of the microbiome between CMV-positive and CMV-negative groups. Diversity analysis and difference analysis showed that there was no significant difference between the CMV-positive group and the CMV-negative group, as shown in **Supplementary Figure 2**. Microbial analysis showed significant differences in top-20 species (such as *Pneumocystis jirovecii* and *Veillonella parvula*) and other species, as shown in **Figure 4**. Our results indicated that virus especially CMV may affect the microbial composition of the lungs of SOT recipients, especially the proportion of *Pneumocystis jirovecii*.

## Discussion

In this study, we explored virus detected in respiratory sample of 43 solid organ transplant recipients by mNGS. Viruses were detected in the bronchoalveolar lavage fluid (BALF) of 26 recipients. The most common type was CMV. The results showed that virus was associated with increased mortality in SOT recipients, and CMV may be the main virus. In addition, our results indicated that the virus may affect the composition of lung microbial species in solid organ transplant recipients.

Unfortunately, although there are differences in mortality between the virus-positive group and the virus-negative group, the survival curve P value did not reach significance (p = 0.0828), which may be related to a small sample size. Previous studies have shown that CMV can increase mortality in SOT recipients (Beam et al., 2016; Teira et al., 2016; Haidar et al., 2020). In this study, our results are consistent with the results of previous studies. In addition, the mNGS detection used in this study can reveal the presence of other viruses in the samples other than CMV. Other viruses detected in this study include parvovirus (TTV), BK polyoma virus (BKV), Epstein-Barr virus (EBV), human parvovirus B19, human herpes virus 7 (HHV7), herpes simplex virus 1 (HSV1), and varicella-zoster virus. Bailey et al. (2019) retrospectively analyzed 39 studies on viral infections in lung transplantation. The most common ones related to mortality were RSV and adenovirus (AdV). Because of the high recipient mortality, it could not be determined whether other viruses were related to mortality (Bailey et al., 2019). Zanella et al. (2020) analyzed viruses other than CMV and EBV in the blood of SOT and hematopoietic stem cell transplant recipients. Hill et al. (2017) studied co-infection with double-stranded DNA viruses such as EBV, CMV, HHV-6, AdV, and BKV. Results showed that co-infection with the increased mortality, but the impact of a single virus was still unknown (Hill et al., 2017). Combining previous studies and our results, we believe that CMV infection is the most important factor in viral infection in SOT recipients, and it may be responsible for the increase in mortality. Whether other single viruses increase mortality still needs further research for clarification.

Due to the immunosuppressive state, the infection of SOT recipients may be multi-pathogenic, and sometimes it is difficult to define a major pathogen. The 8 deaths in this study were all related to infection, of which at least one virus was detected in 6 cases, but in most cases the virus could not be considered as the main infectious agent. In order to check whether the death is related to other factors, we compared the age, BMI, and the proportions of chronic disease between the death group and the survival group, and the results showed no difference. Our results indicate that the virus is associated with a worse prognosis, whether or not it is the main source of infection. Previous related studies have judged the virus as the main pathogen and found that it leads to a worse prognosis. Our results indicate that the presence of the virus may be a marker of poor prognosis.

In this study, why viruses that are not considered as the main pathogens are also related to high mortality may be a topic of our concern. In many cases, the presence of the virus, such as TTV, is considered to be related to the patient’s low immunity (Focosi et al., 2010; Görzer et al., 2014). One hypothesis is that the virus-positive recipients in this study may have a worse immune status than the virus-negative recipients. But whether a worse immune status is related to a worse prognosis also depends on the type of virus that appears, such as CMV or TTV. Although CMV is weakly pathogenic, infection occurs occasionally, while TTV is temporarily not considered to be the pathogen. This may be the reason that whether TTV is positive in this study is not related to the prognosis. This study also showed that the virus changed the lung microbiome, and the relationship between the immune status and the intestinal microbiome has been reported. Another hypothesis about virus is associated with worse prognosis is that the virus may affect the recipient’s prognosis by affecting the lung microbiome. Unfortunately, there are no accurate quantitative indicators that directly reflect the immune status of the recipient.

mNGS is the main detection technique in this study. At present, there are fewer methods to detect viruses, and there are fewer types of viruses that can be detected. mNGS can cover almost all viruses except RNA viruses, which can be detected by mNGS in the RNA process. Therefore, mNGS is a very effective method for multi-virus detection. The virus was detected in 26 of the 43 recipients in this study, with as many as 8 types of viruses. Each recipient had undergone CMV and EBV serological testing on admission, now the data of antibody testing is added in **Supplementary Table 1**. Given the accuracy of virus detection by mNGS has been proved in several previous studies (Miao et al., 2018; Han et al., 2019), that the difference of results between antibody testing and mNGS is due to the sensitivity and specificity of the methods. In addition to viruses, mNGS can simultaneously detect bacteria, fungi and other pathogens. mNGS is the best detection method for microorganisms that often coexist with viruses, such as PJP, which is difficult to cultivate and the positive rate of other detection methods is extremely low. mNGS is a revolutionary technology in pathogen detection. Our results also showed the advantages of mNGS in the detection of viruses and other microorganisms in SOT recipients.

There have been some studies on the microbiome in SOT recipients, but most of them focused on the intestinal flora (Sepulveda et al., 2019; Chong and Koh, 2020), and the research on the lung microbiome is mostly related to lung transplantation (Mitchell, 2019). Our study consisted of a comprehensive analysis of the lung microbiome in various types of SOT recipients, including cases of kidney, lung, and liver transplantation. Our results showed that compared with the corresponding negative group, PJP was significantly different in the virus-positive and CMV-positive groups. It is believed that CMV is often accompanied by PJP. The results of a study involving 52 PJP-infected kidney transplant recipients showed that the CMV co-infection group (14/52, 26.9%) had increased disease severity and risk of transplant failure. Co-infection with PJP and CMV also increased mortality, but the difference was not significant (21.4% vs. 10.5%, p = 0.370) (Lee et al., 2020). In another retrospective study including 70 recipients with non-HIV-infected PJP-positive pneumonia, pulmonary CMV infection rates were 54.3% (38/70), and there was no significant difference in mortality between those with and without co-infection (Yu et al., 2017). In our study, 71.4% (10/14) of CMV-positive cases were associated with PJP infection. The mortality of the PJP-free group appeared to be higher than that of the PJP group (2/4, 50% vs. 3/10, 30%). Among the enrolled recipients in our study, the proportion of PJP infection was relatively higher. A review about the medical history of all recipients revealed that it may be related to irregular prophylaxis. Most patients discontinue the drug by themselves due to digestive symptoms, such as nausea and vomiting or renal damage. Our results demonstrate the high degree of co-infection with CMV and PJP in SOT recipients, but co-infection with PJP may not increase the mortality of CMV-positive recipients.

In addition to PJP, our results also showed that virus affected the proportions of other microbes in the respiratory tract. For example, the proportion of *Moraxella osloensis*, one of the top 20 species in lung microbiology, decreased in the virus-positive group, and the proportion of *Veillonella parvula*, also one of the top 20 species in lung microbiology, decreased in the CMV-positive group. *Moraxella osloensis* is an aerobic gram-negative bacterium that is saprophytic on human skin and mucous membranes. It is also considered to be part of the normal flora of the human respiratory tract. The reported diseases caused by *Moraxella osloensis* infection include endocarditis, meningitis, osteomyelitis, septic arthritis, and bacteremia (Shah et al., 2000). *Veillonella parvula* is an anaerobic gram-negative bacterium and is considered to be a common bacterium in the oral cavity, gastrointestinal tract, and vagina. It has been reported as a pathogen related to meningitis, periodontitis, chronic maxillary sinusitis, sinusitis, osteomyelitis, bacteremia, pelvic abscess, and testicular epididymitis (Bhatti and Frank, 2000). Pathogenicity studies of the above two and other background bacteria are lacking, and the impact of their differences in SOT recipients with viral infections needs further research. The structure of the microbiome may be affected by many factors. In our study, the difference of PJP between CMV positive group and negative group is consistent with previous reports and clinical cognition. And there are only 2 or 3 species in the TOP 20 have significant differences, which indicates that there is no extreme fluctuation in the microbiome. These results imply the reliability of our microbiome analysis results.

Our study also has some limitations. Although the difference in survival curves between the virus-positive group and the negative group could be observed, it was not significant due to the small size of samples. For this same reason, only 6 cases were detected in other virus groups except CMV and TTV, which is meaningless in statistics. In this study, we only extracted DNA from BALF samples, so RNA viruses are outside the scope of our research. Three types of transplanted organs were enrolled in this study, but there were only 4 recipients of liver transplantation, no one died and one of them was virus-positive. So, the results of this study are more related to kidney and lung transplantation. Our research showed that virus had similar effects on mortality in kidney and lung transplantation. However, it is well known that the partial pulmonary microbiota of the recipient comes from the donor, which may undergo a complex reconstruction. Therefore, whether the impact of virus on the lung microbiome of lung transplant recipients is different from other SOT transplants needs more study and elucidation. Due to the BALF samples were collected by performing invasively endoscopy, the patients are unable to tolerate multiple collections during infections. Therefore, it is unlikely to monitor the change of microbiome at different time points in the patients. By realizing this difficulty, in this study, one time analysis of the microbiome in respiratory tract may not be able to demonstrate the dynamic changes.

In summary, we studied the impact of virus on mortality and the lung microbiome in SOT recipients. The results showed that virus increased mortality regardless whether it is the main reason of infection, and CMV may be responsible for this. The results indicate that the presence of the virus may be a marker of poor prognosis. The proportion of PJP in virus-positive recipients increased significantly; the co-infection rate with PJP reached 71.4% in CMV-infected recipients. The results suggest that we should attach great importance to the co-infection with PJP when dealing with CMV infection. The mNGS results revealed the viral spectrum of SOT recipient lung infection, which can provide clinical reference. At present, there is a lack of detection methods for viruses, and mNGS has been gradually applied in clinical pathogen diagnosis. Our study explored the advantages of mNGS for viral infection and simultaneous detection of other pathogens. In addition, virus can also change the respiratory microbiome, and the differences in some background bacteria may provide references for subsequent research and clinical management.

## Data Availability Statement

The raw data of mNGS in this study has been uploaded to the SRA database of NCBI, and the data is public. The project number is PRJNA806679.

## Ethics Statement

The studies involving human participants were reviewed and approved by the Medical Ethics Committee of Sichuan Provincial People’s Hospital. The patients/participants provided their written informed consent to participate in this study. Written informed consent was obtained from the parents. All the authors listed have approved the manuscript that is enclosed.

## Author Contributions

All authors contributed to the study conception and design. Research design and approval were completed by YK, XH, and YF. Recipient enrollment and management were completed by LP. Data analysis and the manuscript writing were completed by FW. Review and editing of manuscript were completed by LP, QC, ZX, and YF. Microbiome analysis were completed by ZX. Transplant and data collection were completed by HH, TT, RY, YH, and XZ. All authors contributed to the article and approved the submitted version.

## Conflict of Interest

Authors FW, QC, ZX, and YF are employed by Genoxor Medical Science and Technology Inc.

The remaining authors declare that the research was conducted in the absence of any commercial or financial relationships that could be construed as a potential conflict of interest.

## Publisher’s Note

All claims expressed in this article are solely those of the authors and do not necessarily represent those of their affiliated organizations, or those of the publisher, the editors and the reviewers. Any product that may be evaluated in this article, or claim that may be made by its manufacturer, is not guaranteed or endorsed by the publisher.

## Supplementary Material

Supplementary Figure 1 Lung microbiome of virus and non-virus infectious recipients with solid organ transplantation. **(A)** Lung microbiome in genus level; **(B)** lung microbiome in species level; **(C)** Estimated species richness was calculated as Shannon index, there were significant differences between virus and non-virus; **(D)** anosim analysis of lung microbiome; **(E)** nonmetric multidimensional scaling analysis revealed that the within-group variance is larger than the between-group variance.

Supplementary Figure 2 Lung microbiome of CMV and non-CMV infectious recipients with solid organ transplantation. **(A)** Lung microbiome in genus level; **(B)** lung microbiome in species level; **(C)** Estimated species richness was calculated as Shannon index, there were no significant differences; **(D)** anosim analysis of lung microbiome; **(E)** nonmetric multidimensional scaling analysis revealed that the within-group variance is larger than the between-group variance.
