# Supplementary figures and images for: Whole Genome Profiling of Lung Microbiome in Solid Organ Transplant Recipients Reveals Virus Involved Microecology May Worsen Prognosis
Source: Front Cell Infect Microbiol. 2022 Mar 16;12:863399. doi: 10.3389/fcimb.2022.863399 (PMC8967177; doi:10.3389/fcimb.2022.863399)

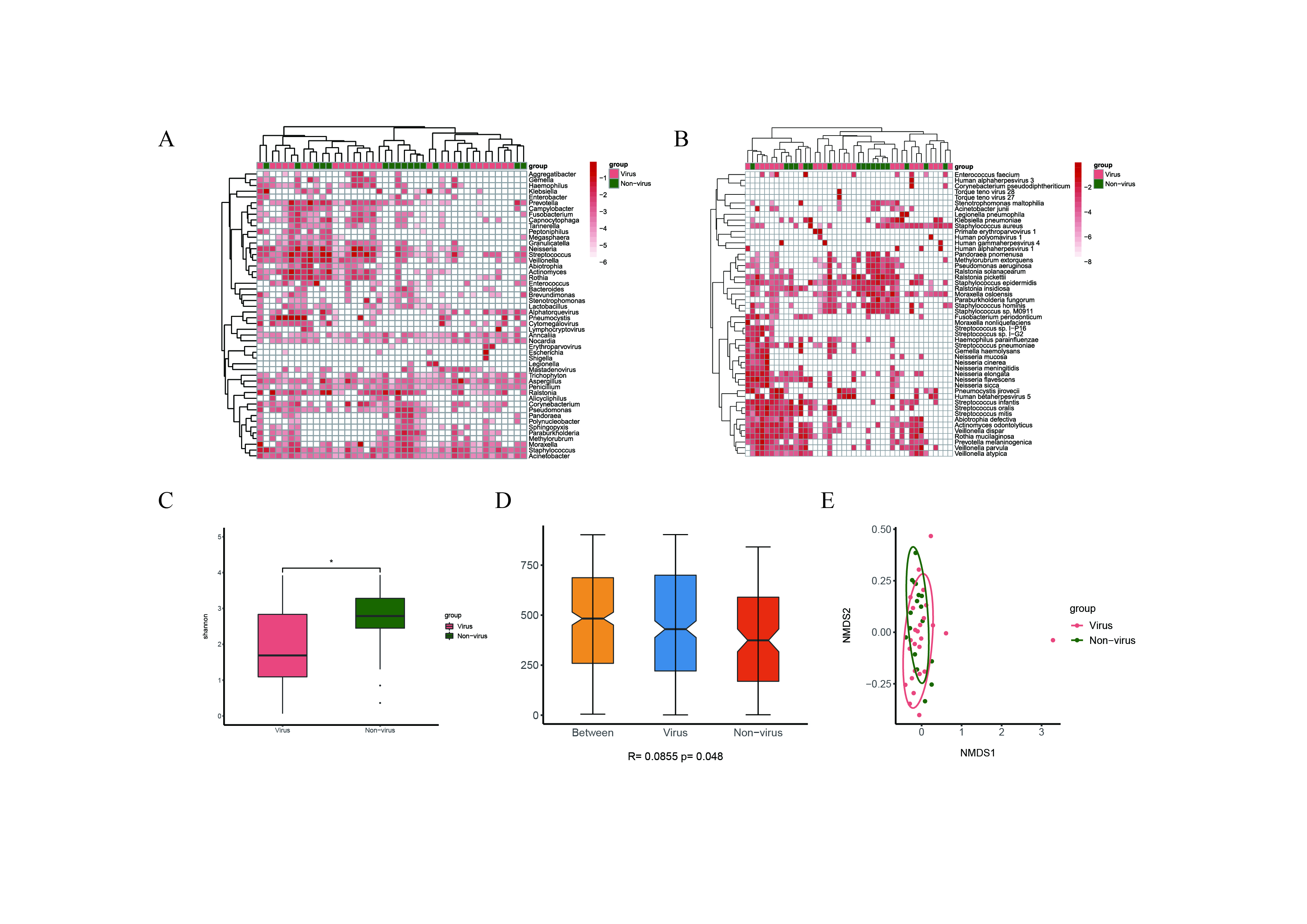

Supplement: Supplementary Figure 1 — Lung microbiome of virus and non-virus infectious recipients with solid organ transplantation. (A) Lung microbiome in genus level; (B) lung microbiome in species level; (C) Estimated species richness was calculated as Shannon index, there were significant differences between virus and non-virus; (D) anosim analysis of lung microbiome; (E) nonmetric multidimensional scaling analysis revealed that the within-group variance is larger than the between-group variance. [file Image_1.jpeg]

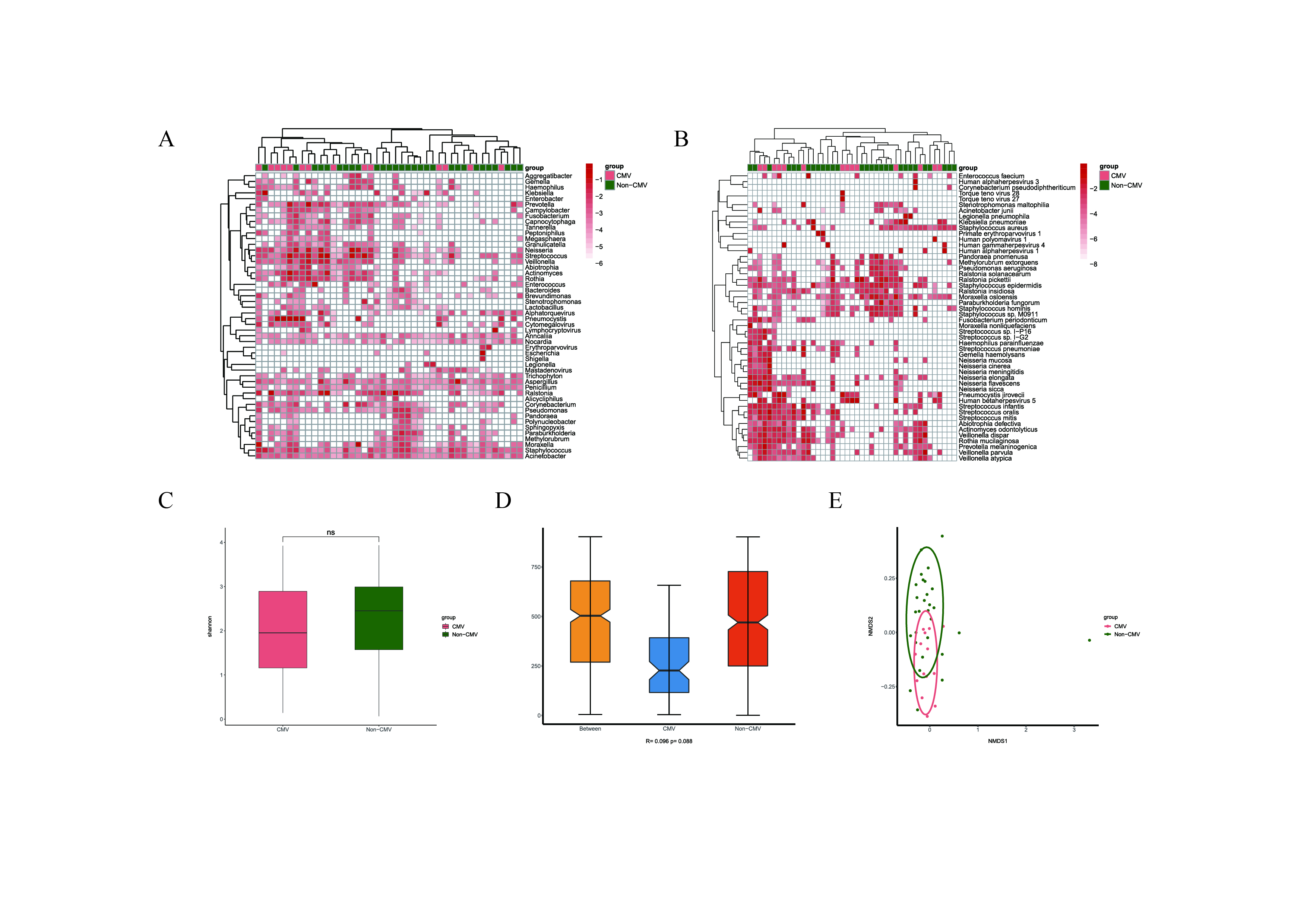

Supplement: Supplementary Figure 2 — Lung microbiome of CMV and non-CMV infectious recipients with solid organ transplantation. (A) Lung microbiome in genus level; (B) lung microbiome in species level; (C) Estimated species richness was calculated as Shannon index, there were no significant differences; (D) anosim analysis of lung microbiome; (E) nonmetric multidimensional scaling analysis revealed that the within-group variance is larger than the between-group variance. [file Image_2.jpeg]
