# Supplementary material for: Whole Genome Profiling of Lung Microbiome in Solid Organ Transplant Recipients Reveals Virus Involved Microecology May Worsen Prognosis
Source: Front Cell Infect Microbiol. 2022 Mar 16;12:863399. doi: 10.3389/fcimb.2022.863399 (PMC8967177; doi:10.3389/fcimb.2022.863399)
Supplement: Supplementary file 4 [file Table_2.docx]

Supplemental table 2. Prognosis in different types of solid organ transplantation.

| Type of solid organ transplantation | The Prognosis of 90 days-no. (%) | Virus | Non-virus |
| --- | --- | --- | --- |
| Kidney | Good | 13(72.2%) | 7(96.03%) |
|  | Poor | 5(27.8%) | 1(3.97%) |
| Lung | Good | 5(71.4%) | 6(100%) |
|  | Poor | 2(28.6%) | 0(0%) |
| Liver | Good | 1(100%) | 3(100%) |
|  | Poor | 0(0%) | 0(0%) |

Good=survival; Poor=died.
