# Supplementary material for: Whole Genome Profiling of Lung Microbiome in Solid Organ Transplant Recipients Reveals Virus Involved Microecology May Worsen Prognosis
Source: Front Cell Infect Microbiol. 2022 Mar 16;12:863399. doi: 10.3389/fcimb.2022.863399 (PMC8967177; doi:10.3389/fcimb.2022.863399)
Supplement: Supplementary file 5 [file Table_3.docx]

| **transplant type** | **CMV** | **TTV** | **BKV** | **EBV** | **B19** | **HHV-7** | **HSV1** | **VZV** | **total positive recipients** |
| --- | --- | --- | --- | --- | --- | --- | --- | --- | --- |
| Kidney | 12 | 6 | 2 | 1 | 1 | 0 | 3 | 1 | 18 |
| Lung | 2 | 2 | 0 | 2 | 0 | 1 | 1 | 0 | 7 |
| Liver | 0 | 0 | 0 | 0 | 1 | 0 | 0 | 0 | 1 |

Supplementary table 3. Viral profiles of the three transplant types individually.
